# *Paecilomyces variotii* xylanase production, purification and characterization with antioxidant xylo-oligosaccharides production

**DOI:** 10.1038/s41598-021-95965-w

**Published:** 2021-08-13

**Authors:** Asmaa Abdella, Samah Ramadan, Ragaa A. Hamouda, Amna A. Saddiq, Nuha M. Alhazmi, Mahmoud A. Al-Saman

**Affiliations:** 1 Department of Industrial Biotechnology, Genetic Engineering and Biotechnology Research Institute, University of Sadat City, Sadat City, Egypt; 2 Department of Botany, Faculty of Science, Mansoura University, Mansoura, Egypt; 3 Department of Biology, Collage of Sciences and Arts Khulais, University of Jeddah, Jeddah, Saudi Arabia; 4 Microbial Biotechnology Department, Genetic Engineering and Biotechnology Research Institute, University of Sadat City, Sadat City, Egypt; 5 College of Science, Department of Biology, University of Jeddah, Jeddah, Saudi Arabia

**Keywords:** Biochemistry, Biotechnology, Microbiology

## Abstract

*Paecilomyces variotii* xylanase was, produced in stirred tank bioreactor with yield of 760 U/mL and purified using 70% ammonium sulfate precipitation and ultra-filtration causing 3.29-fold purification with 34.47% activity recovery. The enzyme purity was analyzed on sodium dodecyl sulfate–polyacrylamide gel electrophoresis (SDS-PAGE) confirming its monomeric nature as single band at 32 KDa. Zymography showed xylan hydrolysis activity at the same band. The purified enzyme had optimum activity at 60 °C and pH 5.0. The pH stability range was 5–9 and the temperature stability was up 70 °C. Fe^2+^and Fe^3+^ exhibited inhibition of xylanase enzyme while Cu^2+^, Ca^2+^, Mg^2+^ and Mn^2+^ stimulated its activity. Mercaptoethanol stimulated its activity; however, Na_2_-EDTA and SDS inhibited its activity. The purified xylanase could hydrolyze beechwood xylan but not carboxymethyl cellulose (CMC), avicel or soluble starch. *Paecilomyces variotii* xylanase K_m_ and V_max_ for beechwood were determined to be 3.33 mg/mL and 5555 U/mg, respectively. The produced xylanase enzyme applied on beech xylan resulted in different types of XOS. The antioxidant activity of xylo-oligosaccharides increased from 15.22 to 70.57% when the extract concentration was increased from 0.1 to 1.5 mg/mL. The enzyme characteristics and kinetic parameters indicated its high efficiency in the hydrolysis of xylan and its potential effectiveness in lignocellulosic hydrolysis and other industrial application. It also suggests the potential of xylanase enzyme for production of XOS from biomass which are useful in food and pharmaceutical industries.

## Introduction

Endo-1,4-β-D-xylanases (EC 3.2.1.8) hydrolyze the β-1,4-xylosidic bonds of the hemicellulose xylan backbone to produce xylo-oligosaccharides (XOS), while β-D-xylosidases hydrolyze the non-reducing ends of XOS to produce xylose^1–3^. According to amino acid sequence and catalytic domain analysis, endoxylanase belong to the glycoside hydrolase (GH) families 10 and 11, however, they also have been mentioned in GH families (5, 7, 8, 16, 26, 30, 43, 52 and 62)^4,5^. Xylanases have been reported to be potentially applicable in industry. They can be used in food industry such as manufacture of bread, dough, juice and beer^6,7^. They can be used in pharmaceutical production as well^2^. Kumar and Satyanarayana^8^ reported the application of xylanases in the paper and pulp industry. Biorefineries also involve xylanases in ethanol production^9^.

Enzymatic hydrolysis of xylan to generate XOS is one important application of xylanase. XOS has a branched structure comprising b-(1,4) bonded 2–7 xylose units and a number of substituent’s such as acetyl groups, uronic acids and arabinose units. The enzymatic production of XOS is beneficial because it does not contain significant amounts of harmful by-products or monosaccharides^10^. XOS has a broad diversity of biological functions, such as promoting the production of beneficial bacteria (bifidobacteria and lactobacilli)^11^, enhancing calcium absorption, reducing colon cancer risk, immune-modulatory and anti-infective properties, blood and skin effects, and antimicrobial actions^12^. XOS aren’t hydrolyzed in the upper part of the gastrointestinal tract and they selectively stimulate the growth or activity of one or a limited number of bacteria in the colon and thus improve health^13^. They also have many essential medicinal uses, such as dermatological applications, antioxidant activity, anti-histaminic and anti-inflammatory properties, immune-modulatory effects, cosmetic applications, cytotoxic activity^14^.

Filamentous fungi were reported to be potential source of enzyme production^15^. *Paecilomyces variotii* is the asexual state of Byssochlamys spectabilis, a member of the Phylum Ascomycota (Family Trichocomaceae)^16^. Paecilomyces has high growth sporulation rates and grows over a wide range of temperatures andsubstrates. As a result, its rapid multiplication ensures viable and affordable development of commercial formulations^17^. Thermophilic fungi are apotential source of thermotolerant enzyme^18^.

There are very few studies on xylanase production by *Paecilomyces variotti*^19^. The present study represents production, purification and characterization of xylanase enzyme produced from *Paecilomyces variotii*. The purified xylanase enzyme was observed to be thermophilic, alkalophilic enzyme which has high specific activity, efficiency, and affinity to xylan substrate compared to other xylanases reported in literature. It also aimed to use the produced xylanase enzyme to produce XOS from beechwood xylan and evaluate its antioxidant properties to be applied in nutraceutical industries.

## Materials and methods

### Microbial strain

This study used *Paecilomyces variotii* NRRL 1115, collected from the Culture Collection of the Agricultural Research Service (ARS) (Peoria, Illinois, USA).

### Preparation of inoculum

Spores reserved in fungal stock solution (20% glycerol, 10% lactose) at − 80 °C were thawed and 150 μL were spread in a Petri dish containing potato dextrose agar (PDA) medium. The plates were incubated at 30 °C for 7 days. The spores were scraped with distilled water, giving a final concentration of approximately 3 × 10^7^ spores/mL.

### Preparation of cell pellets

Cell pellets for seeding submerged fermentation in stirred tanks were prepared by inoculating spores (3 × 10^8^) in a shake-flask containing 150 mL of the preculture medium, which contained only glucose (10 g/L) as the carbon source (no glycerol and wheat bran) and all other medium components as the fermentation medium. After inoculation, the flask was incubated at 30 °C on a rotatory shaker at 200 rpm for 48 h.

### Xylanase production in stirred tank bioreactor

A 3-L stirred-tank reactor (STR) containing 1.5 L of the fermentation medium (g/L) was studied for batch fermentation: 5 glycerol, 3.5 wheat bran, 7.5 corn steep liquor, 1 NaNO_3_, 0.3 K_2_HPO_4_, 0.1 KCl, MgSO_4_·7H_2_O, and 0.01 FeSO_4_·7H_2_O. The bioreactor was inoculated with 150 mL of preformed cell pellets after autoclaving at 121 °C for 30 min and operated at 30 °C with aeration at 2 vvm and agitation at 450 rpm. During the fermentation, the medium pH was not controlled. To control foaming, silicone antifoam 204 (Sigma-Aldrich) was added as required. Broth samples were taken on 8^th^ days and assayed for their β-glucosidase activities after removing the mycelia by centrifugation.

### Purification of xylanase

Ammonium sulphate was added slowly with stirring to the crude enzyme togive 60% saturation at 4 °C, allowed to stir for 60 min, and then allowed to stand for 24 h at 4 °C. After centrifugation at 10,000 rpm for 20 min, supernatant was decanted, and the precipitate was discarded. Ammonium sulfate was added to bring supernatant to 70% saturation under the same conditions. After centrifugation at 10,000 rpm for 20 min, supernatant was decanted, and the precipitate was dissolved in 10 mL, 0.05 M citrate buffer (pH 5), and then dialyzed against the same buffer for 48 h. Then the clear supernatant was concentrated by ultrafiltration using Amicon Ultra centrifugal filters MWCO 10 kDa. The purity was checked using SDS-PAGE.

### SDS-PAGE and zymogram analysis

After ultra-filtration, sodium dodecyl sulfate polyacrylamide gel electrophoresis (SDS-PAGE) zymography was applied^20^. SDS-PAGE was carried out using 10% polyacrylamide in the gel, β-mercaptoethanolwas not added to the samples^21^. On completion of electrophoresis, the gel was cut in two parts. One part was used for Coomassie brilliant blue staining and the other was used for zymography.

Zymography was done according to Kumar et al.^22^. The gel was soaked inside 50 mM sodium phosphate buffer (pH 7.0) containing 25% isopropanol 25% for 30 min at 4 °C. Then, the gel was removed and placed in the same buffer containing 1.0% beechwood xylan substrate at 37 °C for 30 min. 0.1% Congo red and 1 M NaCl were used for staining and de-staining of the gel, respectively. Decolorized bands of the red background indicated xylanase activity.

### Measurement of xylanase and protein activity

Xylanase activity was assayed using xylan from beech wood (Sigma-Aldrich, Egypt) as substrate. 0.95 mL of 1% (xylan) in 0.05 M citrate buffer, (pH 5) was incubated with 0.05 mL of diluted crude enzyme extract at 50 °C for 15 min. Then the reaction was stopped using 0.5 mL of 3,5-dinitrosalicylic acid (DNS) reagent. The contents are boiled on water bath for 5 min. The absorbance was measured at 575 nm after cooling. The absorbance was compared to that of a substrate control1^23^. One international unit (IU) of xylanase activity is defined as the amount of enzyme catalyzing the release of 1 μmol/min of reducing sugar equivalent to xylose under the specified assay condition^24^. Using a protein assay kit (BioRad Laboratories, USA) with bovine serum albumin as the standard, the total protein concentration was evaluated^25^.

### Characterization of xylanase

The molecular weight of xylanase was confirmed by comparison with standard protein markers (Blue stain protein ladder, (20–245) KDa, Gold Biotechnology, USA) separated by SDS-PAGE. To determine optimum temperature, xylanase activity was measured attemperatures ranging from 30 °C to 80 °C in 0.05 M citrate buffer at pH 5. To determine the optimum pH, xylanase activity was measured at 50 °C citrate buffer at pH 3 to 6 and phosphate buffer at pH 7 and 8. Temperature stability was determined by measuring the residual activity after incubating the enzyme in 0.05 M citrate buffer pH 5 at various temperatures (30–80 °C) for 30 min. The pH stability was determined by measuring the remaining activity after incubating the enzyme in series of buffer at pH range of 3 to 9 at 4 °C for 24 h. Effect of metals on the activity of purified xylanase was measured by incubating the enzyme with 5 mM of various metal ions (Zn^2+^, Ca^2+^, K^+^, Mg^2+^, Mn^2+^, Cu^2+^, Na^+^, Co^2+^) in 0.05 M pH 5 citrate buffer for 60 min at 30 °C. Then, the residual enzymatic activity was measured. Effect of other chemicals on the activity of purified xylanase was measured by incubating the enzyme with 5 mM of the following compounds (Na2-EDTA, SDS, urea, dimethylsulfoxide (DMSO), mercaptoethanol and citric acid) in the 0.05 M citrate buffer (pH 5) for 60 min at (30 °C). Then, the residual enzymatic activity was measured.

Substrate specificity for purified xylanase was investigated by incubating the enzyme with a 1% (w/v) of the following polysaccharides (beechwood xylan, CM-cellulose, Avicel, pectin and starch) in 0.05 M pH 5 citrate buffer for 60 min at 30 °C. The initial hydrolysis rate of beechwood xylan at different substrate concentrations (1, 2, 5, 10 and 20 mg/mL) prepared in 0.05 M citrate buffer, pH 5.0 at 50 °C was used to study the enzyme kinetics. The Michaelis constant (K_m_) and maximum velocity (V_max_) values were calculated according to Lineweaver and Burk by linear regression from double-reciprocal plots^26^.

### Analysis of xylo-oligosaccharides produced by xylanase enzyme hydrolysis of xylan

Fifty µL of xylanase was incubated with 1% w/v beechwood xylan in 0.05 M citrate buffer (pH 5) at 55 °C. Aliquots were withdrawn at intervals and boiled for 5 min. Control without enzyme was performed in parallel. Samples were analyzed by HPLC (HPLC, Shimadzu Class-VPV 5.03 (Kyoto, Japan) on an HPX-87H column (300 mm × 7.8 mm). The eluent was HPLC grade DI-water with a flow rate of 0.5 mL/min at 70 °C. Sugars were detected by a refractive index detector (Shodex, RI-101). Xylo-oligosaccharides (xylobiose, X2; xylotriose, X3; xylotetraose, X4; xylopentaose, X5; xylohexaose, X6) and xylose were used as standards for the analysis of the reaction products^27^.

### Antioxidant activity of xylo-oligosaccharides

Using the scavenging effect of radicals on DPPH, antioxidant activity can be monitored. 1 mL of the beechwood hydrolysis products (xylo-oligosaccharides) was mixed with 1 mL of 0.04 mg/mL DPPH solution. The reaction was monitored after 15 min. Absorbance at 517 nm was used to calculate radical scavenging activity (% of inhibition) with the formula.

Inhibition (%) = 1 – Ab_sample_ − Ab_blank_/Ab_control_ − Ab_blank_ × 100.

where Ab sample was the absorbance of the reaction in presence of sample (sample + DPPH solution), Ab blank was the absorbance of the blank for each sample dilution (sample + DPPH solvent) and Ab control was the absorbance of control reaction (sample solvent + DPPH solution). Then, this value obtained for every concentration was plotted to obtain IC50 values in each time point^28^.

### Statistical analysis

All data were subjected to analysis of variance (ANOVA). Three samples of each item were analyzed and the main values as well as the SD were given. Significance of the variable mean differences was determined using Duncan’s multiple range tests (p ≤ 0.05). All analyses were carried out using SPSS 16 software.

### Ethical statements

The manuscript does not contain experiments using human study.

## Results and discussion

### Production and purification of xylanase enzyme

Table 1 shows that the activity of xylanase enzyme produced in stirred tank bioreactor was 760 U/mL. The enzyme was purified by precipitation using 70% saturated ammonium sulfate solution. The precipitated enzyme was concentrated by ultra-filtration (Amicon Ultra-3 kDa, Millipore). The purification protocol led to a 3.29-fold increase in purity with 34.47% xylanase yield (recovery). The specific enzyme activity of the purified enzyme fraction was 3968 U/mg protein. It is one of the highest specific activities ever reported for xylanase^29–32^.

**Table 1:** Chart for the purification of xylanase enzyme.

Fraction type	Volumetric activity (U/mL)	Protein conc (mg/mL)	Total volume (mL)	Total activity (U)	Total protein (mg)	Specific activity (U/mg)	Purification folds	Recovery (%)
Crude enzyme	760	0.63	100	76,000	63	1206	1	100
70% Ammonium sulphate	2560	0.96	20	51,200	19.2	2666	2.210	67
Ultra-filtration	5239	1.32	5	26,195	6.6	3968	3.29	34

### Characterization of xylanase

#### Determination of the molecular weight

**Figure 1:**
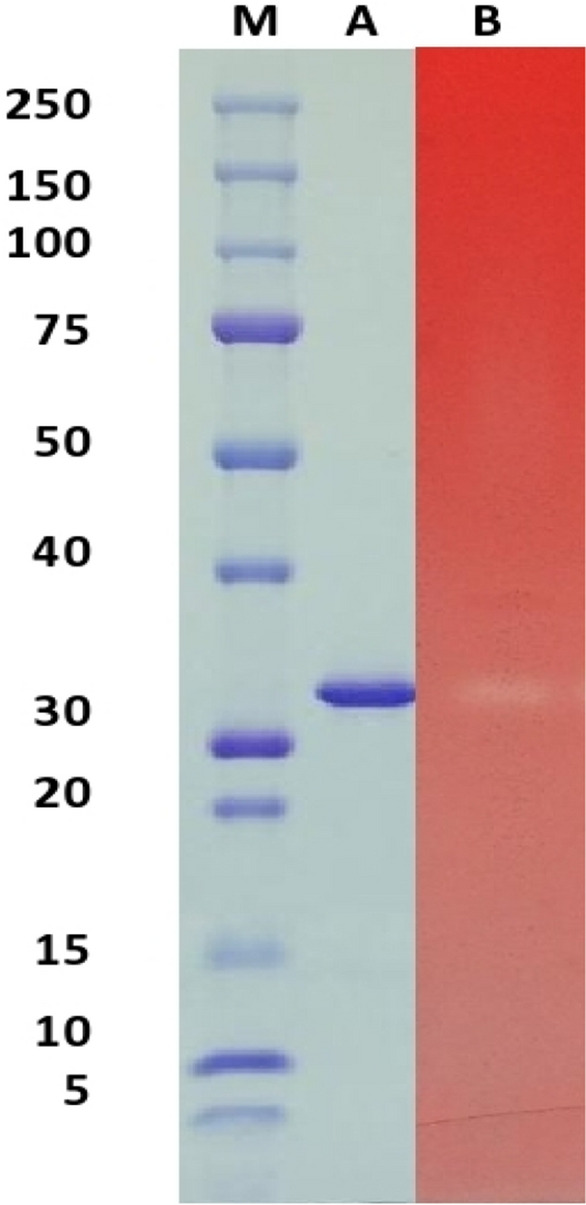
Cropped SDS-PAGE and zymogram of xylanase enzyme produced by Paecilomyces variotti**. **Lane M: Standard protein marker (250, 150, 100, 75, 50, 40, 30, 20, 15, 10, 5). Lane A: SDS-PAGE of the partially purified xylanase enzyme. Lane B: Zymogram of the partially purified xylanase enzyme. (Full-length gels are presented in supplementary figures S1 and S2).

Purified xylanase migrated as a single band on SDS-PAGE suggesting that the purified xylanase was a monomer consisting of a single polypeptide chain. The molecular weight of the xylanase was 32 kDa. The activity of purified xylanase was confirmed through zymography, which showed decolorization of the red background at 32 kDa, which confirmed the enzyme activity at that band (Fig. 1).

According to (Wang et al.,)^33^, who reported that low molecular weight xylanase is at the range of 21–34 kDa, this xylanase is considered to low molecular weight for xylanases. The low molecular weight of our purified xylanase was identical to the enzyme reported from an *Aspergillus mutant* strain^34^, *Bacillus subtilis*^35^, and *Trichoderma inhamatum*^9^. A higher molecular weight xylanase was reported for *Bacillus pumilus*^36^, for *Bacillus* sp. GRE7^37^ and for *Paenibacillus campinasensis* BL11^38^. For the pulp and paper industry, low molecular weight xylanases are favored as they can penetrate pulp fibers more easily than higher molecular weight^39^.

### Effect of temperature on xylanase activity and stability

**Figure 2:**
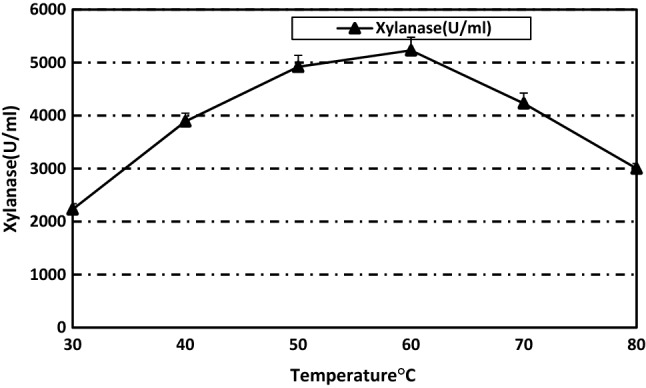
Temperature optima of the purified xylanase from *Paecilomyces variotti.*

The enzyme activity increased as temperature increased reaching a maximum activity at 60 °C, then activity declined at 70 and 80 °C, reaching 57% of its maximum activity at 80 °C (Fig. 2). The observed optimum temperature lies in the same range (50–60 °C) of most reported xylanases^35^.

**Figure 3:**
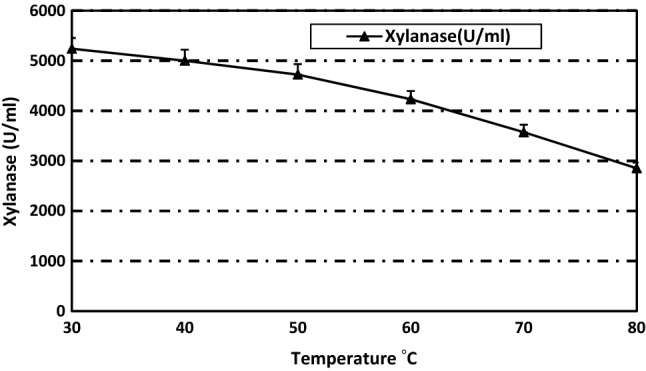
Thermostability of the purified xylanase from *Paecilomyces variotti.*

Regarding thermal stability, 80% of enzyme activity was retained after incubation for 30 min at 60 °C. Only 68% and 55% of activity was retained after incubation for 30 min at 70 and 80 °C, respectively (Fig. 3). Our strain is relatively stable compared to xylanases from other *Aspergillus* species. Xylanase purified from *A. phoenicis*^40^ and *A. giganteus*^41^ exhibited half-lifes of only 25 and 13 min at 50 °C, respectively. Thermal stability is correlated to intermolecular bonds such as hydrogen and disulfide bonds and molecular interactions such as electrostatic and hydrophobic interactions. Studying these stabilizing factors is beneficial in re-engineering of mesophilic enzymes to more stable a enzymes^42^. Temperature stability is required in industrial applications, especially in biomass hydrolysis, which is carried out under high temperature^43^.

### Effect of pH on xylanase activity and stability

**Figure 4:**
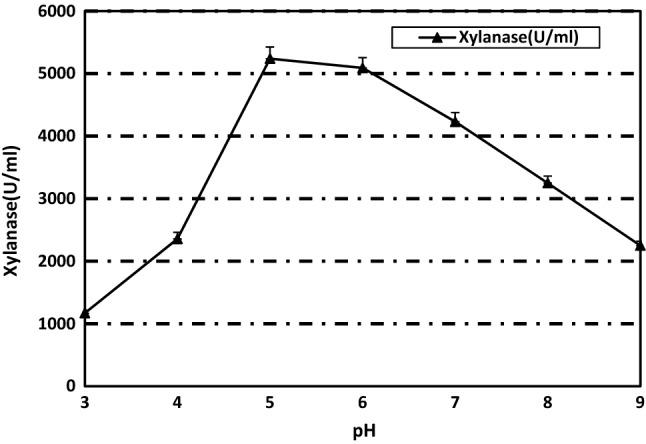
pH optima of the purified xylanase from *Paecilomyces variotti.*

The pH range of 5 to 6 was suitable for enzyme activity with optimum at pH 5 (Fig. 4) nearly similar to *A. kawachii* (pH 5.5)^44^, *A. nidulans* (pH 6.0)^45^ and *A. foetidus* (pH 5.3)^3^. This result confirmed the suitability of *P. variotii* xylanase for use in juice manufacture in which acidic pH is favorable^46^.

**Figure 5:**
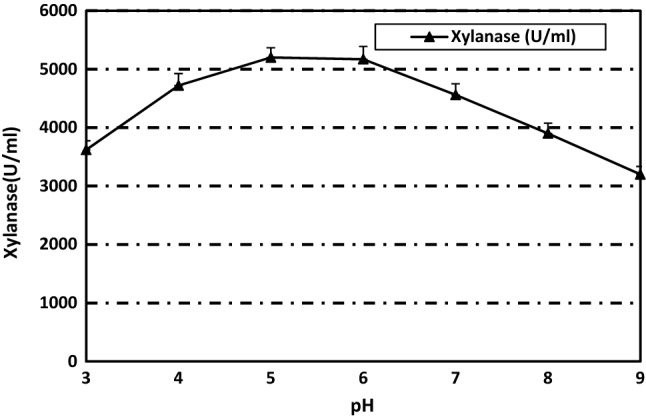
pH stability of the purified xylanase from *Paecilomyces variotti.*

Xylanase enzyme retained 100% activity at pH 5–6, while 87%, 75% and 62% of activity was retained when assayed at pH 7, 8 and 9 respectively, after 24 h incubation (Fig. 5). The pH stability of xylanase from Aspergillus were different it ranged from pH (2 to 7) in case of *A. ochraceus*^47^, (4.5 to 6) in case of *A. niveus*, pH (6.0 to 8.0) in case of *A. fumigatus*^48^ and pH (7 to 9) in case of *A. carneus* M34^49^. The pulp bleaching process is usually carried out in a high temperature (60–80 °C) and high pH (8–10) atmosphere^50^, therefore, the xylanases for this application are required to be thermophilic, thermostable, alkaliphilic, and alkali-stable^51^.

### Effect of metals on xylanase activity

Solution of different metal ions at 5 mM concentration was added to purified xylanase and their effects on xylanase activity are summarized in Table 2. Xylanase activity increased by 32% with Cu^2+^, 20% with Mn^2+^, 15% with Mg^2+^, and 13% with Ca^2+^; similar increases in activity in the presence of Cu^2+^ have been reported for xylanase purified from *A. niger*^30^ and *A. ficuum*^52^. In contrast, xylanases from *A. giganteus*^53^, *Sorangium cellulosum*^32^ and *Geobacillus thermoleovorans*^54^ were inhibited by copper. Enhancement of activity by Mn^2+^ was also reported for PXII-1 xylanase purified from *A. awamori* PXII-1^55^ and for Xyn11NX xylanase purified from *Nesterenkonia xinjiangensis*^56^. (Hmida-Sayari et al.,^57^) also reported that Mg^2+^ improved the xylanase activity purified from *A. niger* by 12%. This may be due to structural stability induced by Mg^2+^^58^. Increases in activity in the presence of Ca^2^^+^ was also reported for xylanase purified from *A. niger*^30^ (Elgharbi et al., 2015). These metals may serve as a cofactor in the enzyme–substrate reaction^6,59,60^. Calcium also protects xylanase from proteinase inactivation and thermal unfolding^61^.

**Table 2:** Effect of metals ions on activity of xylanase enzyme.

Metal ions	Relative activity %
Cu^2+^	132
Ca^2+^	113
Co^2+^	92
Mg^2+^	115
Mn^2+^	120
Zn^2+^	89
K^+^	93
Fe^2+^	63
Fe^3+^	52
Na^+^	97

The metal ions Co^2+^, Zn^2+^, K^+^, Fe^2+^, Fe^3+^ reduced xylanase activity by 8%, 11%, 7%, 37%, and 48%, respectively, at concentrations of 5 mM. The inactivation of xylanase enzyme by the addition of salts of heavy metals such as Fe^2+^ and Fe^3+^ is well known. Yi et al.^62^ reported that Fe^2+^ inhibited activity of xylanases of Aspergillus sp. This inhibition may be due to nonspecific salt formation with the enzyme^36,63^. Metal ion interaction with SH or carboxyl groups will alter protein configuration^64^.

### Effect of chemicals on xylanase activity

Table 3 shows the effects of 5 mM solutions of different chemicals on the enzyme activity. β-mercaptoethanol, due to its reducing activity, enhanced enzyme activity by 55%. Moreira et al.^65^ also reported enhancement of activity by β-mercaptoethanol for xylanase from *A. terrus.* This could be attributed to the prevention of oxidation of the thiol group in the enzyme^66,67^. Vieira Cardoso and Ferreira Filho^68^, also related the protection of cysteine residues from oxidation by mercaptoethanol and this maintain tertiary structure of the active site in *Penicillium citrinum* and A*crophialophor anainiana* xylanases, respectively.

**Table 3:** Effect of chemicals on activity of xylanase enzyme.

Chemical	Relative activity %
DMSO	93
Na_2_-EDTA	75
SDS	23
Citric acid	93
Urea	95
Mercaptoethanol	155

EDTA decreased *P. variotii* xylanase activity by 25% similar to *A. giganteus* xylanase^53^. This may be explained by the enhancement of xylanase activity by some metal ions for xylanase activity, as was observed in this study. Since EDTA is a metal chelator, decreased xylanase activity would be expected in the presence of EDTA^66^. SDS reduced activity by 77%. This large decrease in activity by SDS, which is an anionic surfactant, confirms the importance of hydrophobic interactions for stabilization of enzyme structure^69^. Hmida-Sayari et al.^57^ also reported that SDS decreased activity of xylanase enzyme purified from *A. niger* by 80%.

### Substrate specificity

The greatest activity (5230 U/ml) was observed with beechwood xylan, which contains β-1,4 linkages between D-xylose residues^70^. The residual activity was 7.2% for pectin. This may be due to presence of xylan residues in pectin^71^. The enzyme was completely inactive with starch, avicel and carboxy-methyl cellulose sodium salt as substrates (Table 4). The same results were obtained by Chen et al.,^72^ who also observed that a xylanase enzyme produced from *A. niger* had no activity towards starch, Avicel and carboxy-methyl cellulose. Bai et al.,^73^ also reported that xylanase from Bacillus was inactive towards CMC, pectin, and starch.It has been suggested that shape recognition of the polysaccharide chain conformation plays a role in polysaccharidase speci¢city. Xylanase recognize the threefold helical structure of xylan as substrate and didn’t recognize the flat ribbon like conformation of cellulose^74^.

**Table 4:** Substrate specificity of purified xylanase enzyme.

Substrate	Relative activity (%)
Xylan from beechwood	100
Carboxy methyl cellulose (CMC) sodium salt	0
Avicel	0
Starch	0
Pectin	7.2

### Determination of kinetic parameters of xylanase

**Figure 6:**
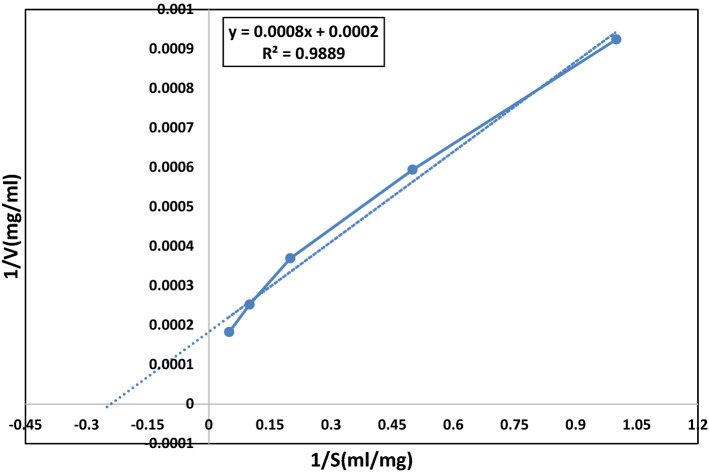
Lineweaver–Burk plot of xylanase enzyme from *Paecilomyces variotti.*

The hydrolytic activity of the purified xylanase was measured using beechwood xylan as a substrate at concentrations of 1, 2, 5, 10 and 20 mg/mL. The xylanase was observed to exhibit Michaelis–Menten Kinetics. The K_m_ and V_max_ values obtained from the Lineweaver–Burk plot for beechwood xylan were 3.33 mg/mL and 5555 U/mg respectively (Fig. 6). Xylanase produced in our study was shown to have low K_m_ 3.33 mg/mL and very high V_max_ 5555 U/mg compared to *A. terrus, A. fumigatus, A. kawachii, Penicillium glabrum, Sorangium cellulosum* and *Saccharopolyspora pathumthaniensis* which have K_m_ (2.09, 3.12, 10, 3.1, 26.5 and 3.92) and V_max_ (640, 2587, 1250, 194,7.89 and 256) respectively^29–32,75^. Table 5 A low K_m_ indicated high affinity to substrate while high V_max_ indicate high enzyme activity. K_m_ and V_max_ values were in the range of reported literature (0.09 to 40.9 mg/L for K_m_) and (0.106 to 10,000 U/mg for V_max_), respectively.

**Table 5:** Comparison of characteristic of xylanase enzymes purified from different microorganisms.

Microorganism	K_m_ Beechwood (mg/ml)	V_max_ (U/mg)	Reference
*Paecilomyces variotti*	3.33	5555	This study
*Aspergillus terrus*	2.09	640	Elgharbi et al.,2015
*Aspergillus fumigatus*	3.12	2587	Amir et al.,2013
*Penicillium glabrum*	3.1	194	Knob et al.,2013
*Sorangium cellulosum*	26.5	7.89	Wang et al.,2012

### Analysis of xylooligosaccharides produced by xylanase enzyme hydrolysis of xylan

**Figure 7:**
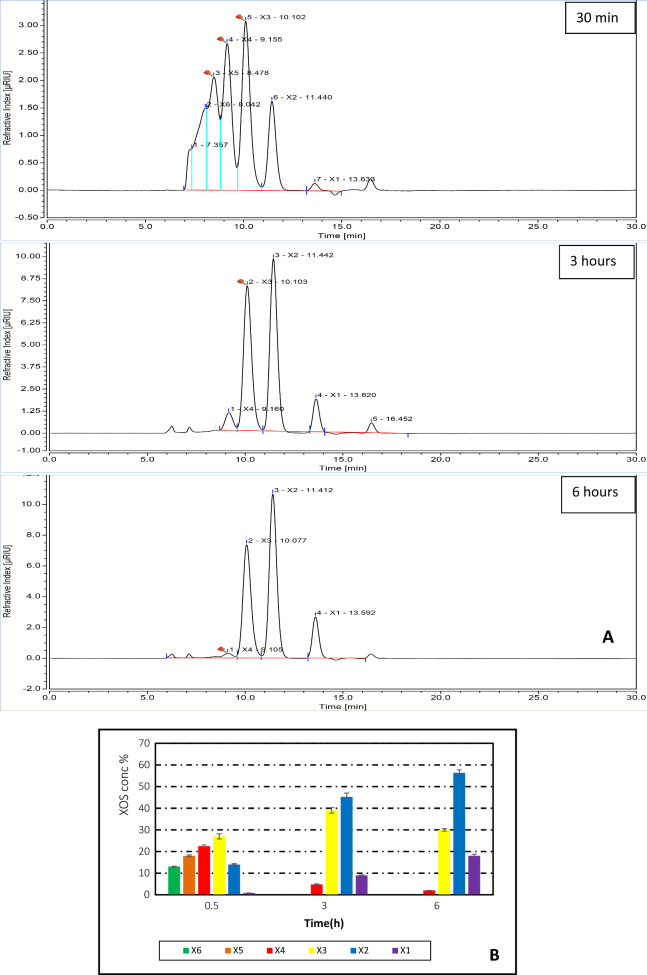
(**A**): HPLC analysis of xylo-oligosaccharides (XOS) of beechwood xylan with purified xylanase enzyme produced by *Paecilomyces variotti*; (**B**) effect of reaction time on enzymatic hydrolysis and relative concentrations of XOS. ^*^ *Xylose (X1), xylobiose (X2), xylotriose (X3), xylotetraose (X4), xylopentaose (X5), xylohexose (X6).*

The hydrolytic constituents of insoluble beechwood xylan incubated with xylanase were identified to give better understanding of the mode of action of purified xylanase. The concentrations of XOS from hydrolysis for different times are depicted in Fig. 7. The XOS produced with xylan hydrolysis by xylanase secreted by *P. variotti* mainly were composed of xylobiose (X2), xylotriose (X3), and xylotetrose(X4), together with a small amount of xylopentaose (X5) and xylohexose (X6) and xylose (X1).

The accumulation of XOS in the hydrolysate was about 0.8% X1, 14% X2, 27% X3, 23% X4, 18% X5 and 13% X6 after incubation for 0.5 h.The fast accumulation of X3 and X4 during the initial 0.5 h was possibly due to the preferential action of xylanase enzyme on xylan chain termini. The finding was consistent with Lin et al.,^76^ who stated that higher levels of X3 and X4 were found at the initial stage of hydrolysis reactions. After 3 h, X1 (9%), X2 (45%) and X3 (39%) contents had increased and the content of X4 (5%) decreased. While X5 and X6 disappeared. The disappearance of X5 and X6 was due to rapid hydrolysis of X5 and X6 into smaller oligosaccharides immediately after release. The same findings were obtained by Akpinar et al.,^77^ who stated that by increasing time of hydrolysis, concentration of XOS with a higher degree of polymerization (DP > 5) decreased. After 6 h, X1 (18%), X2 (56%) had increased, while X3 (30%) and X4 (2%) contents decreased. Since xylose concentration was less than the concentrations of XOS, especially xylotriose and xylobiose, it can be concluding that the purified xylanase was an endo-xylanase that randomly cleaves the internal glycosidic linkages in xylan as a substrate^78^.

In the present study, the XOS obtained were principally composed of xylobiose, xylotriose, and xylotetraose. Therefore, in biotechnological applications, xylanase may have potential applications, because these applications rely on its ability to solubilize hemicellulose rather than complete hydrolysis to xylose.

### Antioxidant activity ofXylooligosaccharides

The DPPH radical-scavenging ability of XOS mixtures obtained from enzymatic hydrolysis of xylan using xylanase enzyme after 6 h of enzymatic hydrolysis was shown in Table 6. Xylo-oligosaccharides have a dose dependent antioxidant activity. The antioxidant activity increased with increasing concentration of XOS mixtures. Based on statistical analysis; the maximum scavenging percentage (70.57%) was observed using the concentration of 2 mg/mL (p < 0.05). moreover, the concentration of 0. 1 mg/mL gave the lowest scavenging potency with an average of 15.22% (P < 0.05).The same results were obtained by Bian et al.,^79^ who stated that increasing XOS concentration, increased antioxidant activity. IC_50_, of XOS mixture obtained after 6 h enzymatic hydrolysis using xylanase enzymes was 0.7154 mg/mL.

**Table 6:** Radical scavenging activity of xylo-oligosaccharides at different concentrations and hydrolysis time toward DPPH.

Conc (mg/mL)	Hydrolysis time (h)			
	0.5	3	6	Conc mean ± SE
2	53.54 ± 0.05^a^*	76.62 ± 0.09^**a**^	81.54 ± 0.06^**a**^	70.57 ± 12.21^**a**^
1.5	41.89 ± 0.05^**b**^	71.92 ± 0.06^**b**^	75.27 ± 0.07^**b**^	63.02 ± 15.01^**b**^
1	29.24 ± 0.02^**c**^	61.46 ± 0.04^**c**^	68.22 ± 0.05^**c**^	52.97 ± 17.01^**c**^
0.5	25.86 ± 0.04^**d**^	37.24 ± 0.03^**d**^	43.08 ± 0.06^**d**^	35.39 ± 07.15^**d**^
0.2	22.89 ± 0.03^**d**^	26.27 ± 0.03^**e**^	30.38 ± 0.07^**e**^	26.51 ± 03.06^**e**^
0.1	00.00	21.48 ± 0.04f.	24.18 ± 0.06f.	15.22 ± 10.82f.
Time mean ± SE	28.90 ± 16.62^**c**^	49.16 ± 21.81^**b**^	53.78 ± 22.28^**a**^	

*Radical scavenging activity given as percentage inhibition.

The percentage inhibition of the standard ascorbic acid was 100%. Values are means of three replicates and the relative standard deviations ˂1%. Means followed by the same letter(s) within a column are not significantly different (p ≤ 0.05) according to Duncan’s multiple range test.

XOS has a branched structure comprising b-(1,4) bonded 2–7 xylose units and a number of substituents such as acetyl groups, uronic acids and arabinose units^80^. Structure of XOS vary in degree of polymerization (DP), monomeric units, and types of linkages depending on the source of xylan used for XOS production^81^. In addition to xylose residues, xylan is usually found in combination with other side groups such as α-D-glucopyranosyl uronic acid or its 4-O-methyl derivative, acetyl groups, or arabinofuranosyl residues^82^. Rao and Muralikrishna^83^ demonstrated that the existence of uronyl or acetyl sugars imparts strong antioxidant activity to polysaccharides. Carboxyl groups have also been reported to enhance the antioxidant function of polysaccharides^84^. The antioxidant mechanism may be also due to the supply of hydrogen by polysaccharides, which combines with radicals and forms a stable radical to terminate the radical chain reaction. The other possibility is that polysaccharides can combine with the radical ions which are necessary for radical chain reaction; then the reaction is terminated^85^. These findings showed that beech xylan XOS has outstanding radical-scavenging activity of DPPH and can be useful against oxidative damage.

## Conclusion

Overall, *Paecilomyces variotii* strain produced low molecular weight thermophilic alkalophilic endoxylanase enzyme. It exhibited high stability at relatively high temperature and alkaline pH. It has a high affinity for xylan, very high V_max_ and one of the highest specific activities ever reported for xylanase. The properties of the purified xylanase make it an ideal candidate in hydrolysis of lignocellulosic materials and other industries. It also suggests the potential of xylanase enzyme for production of XOS with potent antioxidant activities which are useful in food and pharmaceutical industries.

## Supplementary Information

Supplementary Information.

## References

[1] Subramaniyan S, Prema P (2002). Biotechnology of microbial xylanases: Enzymology, molecular biology, and application. Crit. Rev. Biotechnol..

[2] Topakas E, Katapodis P, Kekos D, Macris BJ, Christakopoulos P (2003). Production and partial characterization of xylanase by *Sporotrichum thermophile* under solid-state fermentation. World J. Microbiol. Biotechnol..

[3] Shah AR, Shah RK, Madamwar D (2006). Improvement of the quality of whole wheat bread by supplementation of xylanase from *Aspergillus foetidus*. Bioresour. Technol..

[4] Verma D, Anand A, Satyanarayana T (2013). Thermostable and alkalistable endoxylanase of the extremely thermophilic bacterium *Geobacillus thermodenitrificans* TSAA1: cloning, expression, characteristics and its applicability in generating xylooligosaccharides and fermentable sugars. Appl. Biochem. Biotechnol..

[5] Uday USP, Choudhury P, Bandyopadhyay TK, Bhunia B (2016). Classification, mode of action and production strategy of xylanase and its application for biofuel production from water hyacinth. Int. J. Biol. Macromol..

[6] Bajaj BK, Manhas K (2012). Production and characterization of xylanase from *Bacillus licheniformis* P11 (C) with potential for fruit juice and bakery industry. Biocatal. Agric. Biotechnol..

[7] Butt MS, Tahir-Nadeem M, Ahmad Z, Sultan MT (2008). Xylanases and their application in baking industry. Food Technol. Biotechnol..

[8] Kumar V, Satyanarayana T (2014). Production of thermo-alkali-stable xylanase by a novel polyextremophilic *Bacillus halodurans* TSEV1 in cane molasses medium and its applicability in making whole wheat bread. Bioprocess Biosyst. Eng..

[9] Silva JPA, Mussatto SI, Roberto IC, Teixeira JA (2012). Fermentation medium and oxygen transfer conditions that maximize the xylose conversion to ethanol by *Pichia stipitis*. Renew. Energy.

[10] Zhao LC, Wang Y, Lin JF, Guo LQ (2012). Adsorption and kinetic behavior of recombinant multifunctional xylanase in hydrolysis of pineapple stem and bagasse and their hemicellulose for Xylo-oligosaccharide production. Bioresour. Technol..

[11] Davani-Davari D (2019). Prebiotics: Definition, types, sources, mechanisms, and clinical applications. Foods.

[12] Sheu WHH, Lee IT, Chen W, Chan YC (2008). Effects of xylooligosaccharides in type 2 diabetes mellitus. J. Nutr. Sci. Vitaminol..

[13] Vázquez MJ, Alonso JL, Domínguez H, Parajó JC (2002). Enzymatic processing of crude xylooligomer solutions obtained by autohydrolysis of Eucalyptus wood. Food Biotechnol..

[14] Akhtar MS, Swamy MK (2018). Anticancer plants: Natural products and biotechnological implements. Anticancer Plants Nat. Prod. Biotechnol. Implements.

[15] Jun H, Kieselbach T, Jönsson LJ (2011). Enzyme production by filamentous fungi: Analysis of the secretome of *Trichoderma reesei* grown on unconventional carbon source. Microb. Cell Fact..

[16] Yazdanpanah L, Mohamadi N (2014). Antifungal activity of clove essential oil from *Syzygium aromaticum* on *Paecilomyces variotii* agent of pistachio dieback. J. Biodivers. Environ. Sci.

[17] Moreno-Gavíra A, Huertas V, Diánez F, Santos M, Sánchez-Montesinos B (2020). Paecilomyces and its importance in the biological control of agricultural pests and diseases. Plants.

[18] Luangsa-ard JJ, Manoch L, Hywel-jones N, Artjariyasripong S, Samson RA (2004). Thermotolerant and thermoresistant paecilomyces and its teleomorphic states isolated from Thai forest and mountain soils. Nat. Sci..

[19] Yang SQ (2006). High-level of xylanase production by the thermophilic *Paecilomyces themophila* J18 on wheat straw in solid-state fermentation. Bioresour. Technol..

[20] Laemmli UK (1970). Cleavage of structura l proteins during the assembly of the head of bacteriop hage. Nature.

[21] Raj A, Kumar S, Singh SK (2013). A highly thermostable xylanase from *Stenotrophomonas maltophilia*: Purification and partial characterization. Enzyme Res..

[22] Kumar S (2017). Purification, characterization and thermostability improvement of xylanase from *Bacillus amyloliquefaciens* and its application in pre-bleaching of kraft pulp. 3 Biotech.

[23] Detns, R. C. *et al.* Use of dinitrosaiicyiic acid reagent for determination of reducing sugar.

[24] Bailey MJ, Biely P, Poutanen K (1992). Interlaboratory testing of methods for assay of xylanase activity. J. Biotechnol..

[25] Bradford MM (1976). Rapid and sensitive method for quantitation of microgram quantities of protein utilizing principle of protein-dye binding. Anal. Biochem..

[26] Lineweaver H, Burk D (1934). The determination of enzyme dissociation constants. J. Am. Chem. Soc..

[27] Lee DS (2012). Characterization and pH-dependent substrate specificity of alkalophilic xylanase from *Bacillus alcalophilus*. J. Ind. Microbiol. Biotechnol..

[28] Al-Saman MA, Abdella A, Mazrou KE, Tayel AA, Irmak S (2019). Antimicrobial and antioxidant activities of different extracts of the peel of kumquat (*Citrus japonica* Thunb). J. Food Meas. Charact..

[29] Amir A, Arif M, Pande V (2013). Purification and characterization of xylanase from *Aspergillus fumigatus* isolated from soil. Afr. J. Biotechnol..

[30] Bejar S, Hmida-Sayari A (2014). Expression of A. niger US368 xylanase in *E. coli*: Purification, characterization and copper activation. Int. J. Biol. Macromol..

[31] Knob A, Beitel SM, Fortkamp D, Terrasan CRF, De Almeida AF (2013). Production, purification, and characterization of a major *penicillium glabrum* xylanase using brewer’s spent grain as substrate. Biomed Res. Int..

[32] Wang SY, Hu W, Lin XY, Wu ZH, Li YZ (2012). A novel cold-active xylanase from the cellulolytic myxobacterium *Sorangium cellulosum* So9733-1: Gene cloning, expression, and enzymatic characterization. Appl. Microbiol. Biotechnol..

[33] Wang SL (2003). Production of xylanases from rice bran by *Streptomyces actuosus* A-151. Enzyme Microb. Technol..

[34] Evstatieva Y, Nikolova D, Ilieva S, Getov L, Savov V (2014). I Identification and characterization of α-amylase and endoxylanase, produced by *Aspergillus Mutant* strains. Biotechnol. Biotechnol..

[35] Sanghi, A., Garg, N., Gupta, V. K., Mittal, A., & Kuhad, R. C. (2010). One-step purification and characterization of cellulase-free xylanase produced by alkalophilic *Bacillus subtilis* ash. *Braz. J. Microbiol. *9416782476, 467–476 (2010). 10.1590/S1517-838220100002000029 PMC3768699 24031518

[36] Menon G, Mody K, Keshri J, Jha B (2010). Isolation, purification, and characterization of haloalkaline xylanase from a marine *Bacillus pumilus* strain, GESF-1. Biotechnol. Bioprocess Eng..

[37] Kiddinamoorthy J, Anceno AJ, Haki GD, Rakshit SK (2008). Production, purification and characterization of Bacillus sp. GRE7 xylanase and its application in eucalyptus Kraft pulp biobleaching. World J. Microbiol. Biotechnol..

[38] Ko CH (2007). *Paenibacillus campinasensis* BL11: A wood material-utilizing bacterial strain isolated from black liquor. Bioresour. Technol..

[39] Walia A, Guleria S, Mehta P, Chauhan A, Parkash J (2017). Microbial xylanases and their industrial application in pulp and paper biobleaching: A review. 3 Biotech.

[40] Chipeta ZA, Du Preez JC, Szakacs G, Christopher L (2005). Xylanase production by fungal strains on spent sulphite liquor. Appl. Microbiol. Biotechnol..

[41] Coelho GD, Carmona EC (2003). Xylanolytic complex from *Aspergillus giganteus*: Production and characterization. J. Basic Microbiol..

[42] Bajaj BK, Sharma P (2011). An alkali-thermotolerant extracellular protease from a newly isolated *Streptomyces* sp. DP2. N. Biotechnol..

[43] Turner P, Mamo G, Karlsson EN (2007). Potential and utilization of thermophiles and thermostable enzymes in biorefining. Microb. Cell Fact..

[44] Ito K, Ogasawara H, Sugimoto T, Ishikawa T (1992). Purification and properties of acid stable Xylanases from *Aspergillus kawachii*. Biosci. Biotechnol. Biochem..

[45] Fernández-Espinar M (1994). Purification, characterization and regulation of the synthesis of an *Aspergillus nidulans* acidic xylanase. Appl. Microbiol. Biotechnol..

[46] Pal A, Khanum F (2011). Purification of xylanase from *Aspergillus niger* DFR-5: Individual and interactive effect of temperature and pH on its stability. Process Biochem..

[47] Betini JHA (2009). Xylanases from *Aspergillus niger*, *Aspergillus niveus* and *Aspergillus ochraceus* produced under solid-state fermentation and their application in cellulose pulp bleaching. Bioprocess Biosyst. Eng..

[48] de Carvalho Peixoto-Nogueira S (2009). Production of xylanase by Aspergilli using alternative carbon sources: application of the crude extract on cellulose pulp biobleaching. J. Ind. Microbiol. Biotechnol..

[49] Fang HY, Chang SM, Lan CH, Fang TJ (2008). Purification and characterization of a xylanase from *Aspergillus carneus* M34 and its potential use in photoprotectant preparation. Process Biochem..

[50] Collins T, Gerday C, Feller G (2005). Xylanases, xylanase families and extremophilic xylanases. FEMS Microbiol. Rev..

[51] Li F, Xie J, Zhang X, Zhao L (2015). Improvement of the optimum pH of *Aspergillus niger* xylanase towards an alkaline pH by site-directed mutagenesis. J. Microbiol. Biotech..

[52] Lu F (2008). Purification and characterization of xylanase from *Aspergillus ficuum* AF-98. Bioresour. Technol..

[53] Fialho MB, Carmona EC (2004). Purification and characterization of xylanases from *Aspergillus giganteus*. Folia Microbiol..

[54] Sharma A, Adhikari S, Satyanarayana T (2007). Alkali-thermostable and cellulase-free xylanase production by an extreme thermophile *Geobacillus thermoleovorans*. World J. Microbiol. Biotechnol..

[55] Teixeira RSS, Siqueira FG, De Souza MV, Filho EXF, Da Silva Bon EP (2010). Purification and characterization studies of a thermostable β-xylanase from *Aspergillus awamori*. J. Ind. Microbiol. Biotechnol..

[56] Kui H (2010). Gene cloning, expression, and characterization of a thermostable xylanase from *Nesterenkonia xinjiangensis* CCTCC AA001025. Appl. Biochem. Biotechnol..

[57] Hmida-Sayari A, Taktek S, Elgharbi F, Bejar S (2012). Biochemical characterization, cloning and molecular modeling of a detergent and organic solvent-stable family 11 xylanase from the newly isolated *Aspergillus niger* US368 strain. Process Biochem..

[58] Manikandan K (2006). Crystal structures of native and xylosaccharide-bound alkali thermostable xylanase from an alkalophilic *Bacillus* sp. NG-27: Structural insights into alkalophilicity and implications for adaptation to polyextreme conditions. Protein Sci..

[59] Lv Z, Yang J, Yuan H (2008). Production, purification and characterization of an alkaliphilic endo-β-1,4-xylanase from a microbial community EMSD5. Enzyme Microb. Technol..

[60] Park I, Cho J (2010). Partial characterization of extracellular xylanolytic activity derived from *Paenibacillus* sp. KIJ1. Afr. J. Microbiol. Res..

[61] Spurway, T. D. *et al.* Calcium protects a mesophilic xylanase from proteinase inactivation and thermal unfolding *. **272**, 17523–17530 (1997). 10.1074/jbc.272.28.17523 9211898

[62] Yi X (2010). Hyperexpression of two *Aspergillus niger* xylanase genes in *Escherichia coli* and characterization of the gene products. Braz. J. Microbiol..

[63] Miao Y, Li J, Xiao Z, Shen Q, Zhang R (2015). Characterization and identification of the xylanolytic enzymes from *Aspergillus fumigatus* Z5. BMC Microbiol..

[64] Nagar S, Mittal A, Kumar D, Gupta VK (2012). Production of alkali tolerant cellulase free xylanase in high levels by *Bacillus pumilus* SV-205. Int. J. Biol. Macromol..

[65] De Souza Moreira LR (2013). Two β-xylanases from *Aspergillus terreus*: Characterization and influence of phenolic compounds on xylanase activity. Fungal Genet. Biol..

[66] Knob A, Terrasan CRF, Carmona EC (2010). β-Xylosidases from filamentous fungi: An overview. World J. Microbiol. Biotechnol..

[67] Dutta T (2007). A novel cellulase free alkaliphilic xylanase from alkali tolerant *Penicillium citrinum*: Production, purification and characterization. Lett. Appl. Microbiol..

[68] Vieira Cardoso OA, Ferreira Filho EX (2003). Purification and characterization of a novel cellulase-free xylanase from *Acrophialophora nainiana*. FEMS Microbiol. Lett..

[69] Zheng H (2012). Isolation, purification, and characterization of a thermostable xylanase from a novel strain, *Paenibacillus campinasensis* G1–1. J. Microbiol. Biotechnol..

[70] Sharma K (2020). acacia xylan as a substitute for commercially available xylan and its application in the production of xylooligosaccharides. ACS Omega.

[71] Filho EXF, Puls J, Coughlan MP (1993). Biochemical characteristics of two endo-beta-1,4-xylanases produced by *Penicillium capsulatum*. J. Ind. Microbiol..

[72] Chen H, Yan X, Liu X, Wang M, Huang H (2006). Purification and characterization of novel bifunctional xylanase xylan III isolated from *Aspergillus niger* A-25. J. Microb. Biotechnol..

[73] Bai W, Xue Y, Zhou C, Ma Y (2015). Cloning, expression, and characterization of a novel alkali-tolerant xylanase from alkaliphilic Bacillus sp. SN5. Biotechnol. Appl. Biochem..

[74] Lo Leggio L (2001). Substrate specificity and subsite mobility in T. aurantiacus xylanase 10A. FEBS Lett..

[75] Qiu J (2016). Residue mutations of xylanase in *Aspergillus kawachii* alter its optimum pH. Microbiol. Res..

[76] Lin YS, Tseng MJ, Lee WC (2011). Production of xylooligosaccharides using immobilized endo-xylanase of *Bacillus halodurans*. Process Biochem..

[77] Akpinar O, Erdogan K, Bostanci S (2009). Enzymatic production of Xylooligosaccharide from selected agricultural wastes. Food Bioprod. Process..

[78] Yang Y (2019). Cooperation of hydrolysis modes among xylanases reveals the mechanism of hemicellulose hydrolysis by *Penicillium chrysogenum* P33. Microb. Cell Fact..

[79] Bian J (2013). Structural features and antioxidant activity of xylooligosaccharides enzymatically produced from sugarcane bagasse. Bioresour. Technol..

[80] Caparrós S, Garrote G, Ariza J, Díaz MJ, López F (2007). Xylooligosaccharides production from *Arundo donax*. J. Agric. Food Chem..

[81] Iliev I, Vasileva T, Bivolarski V, Momchilova A, Ivanova I (2020). Metabolic profiling of xylooligosaccharides by lactobacilli. Polymers.

[82] Aachary AA, Prapulla SG (2011). Xylooligosaccharides (XOS) as an emerging prebiotic: Microbial synthesis, utilization, structural characterization, bioactive properties, and applications. Compr. Rev. Food Sci. Food Saf..

[83] Rao RSP, Muralikrishna G (2006). Water soluble feruloyl arabinoxylans from rice and ragi: Changes upon malting and their consequence on antioxidant activity. Phytochemistry.

[84] Pristov JB, Mitrović A, Spasojević I (2011). A comparative study of antioxidative activities of cell-wall polysaccharides. Carbohydr. Res..

[85] Chen Y, Xie MY, Nie SP, Li C, Wang YX (2008). Purification, composition analysis and antioxidant activity of a polysaccharide from the fruiting bodies of *Ganoderma atrum*. Food Chem..

