# Supplementary material for: Paecilomyces variotii xylanase production, purification and characterization with antioxidant xylo-oligosaccharides production
Source: Sci Rep. 2021 Aug 13;11:16468. doi: 10.1038/s41598-021-95965-w (PMC8363652; doi:10.1038/s41598-021-95965-w)
Supplement: Supplementary file 1 — Supplementary Information. [file 41598_2021_95965_MOESM1_ESM.docx]

**Supplementary figures**

*
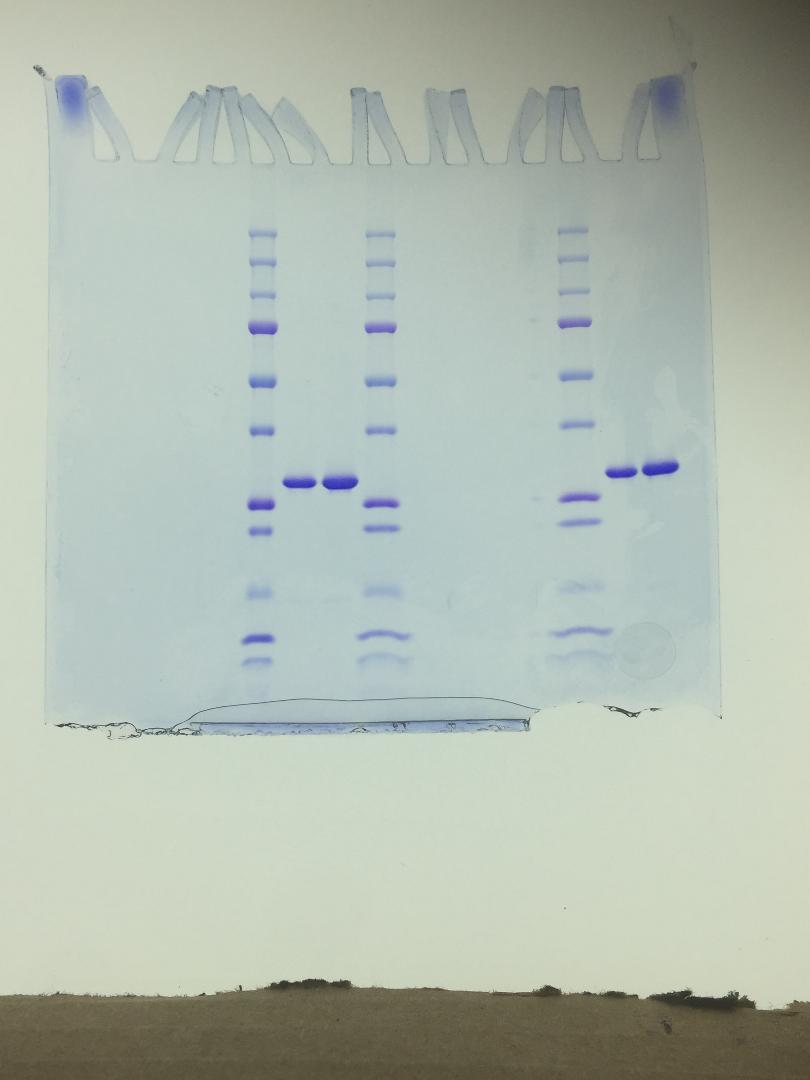
*

**Figure S1**

*SDS-PAGE of the partially purified xylanase enzyme* against *standard protein marker (250, 150, 100, 75, 50, 40, 30, 20, 15, 10, 5)*


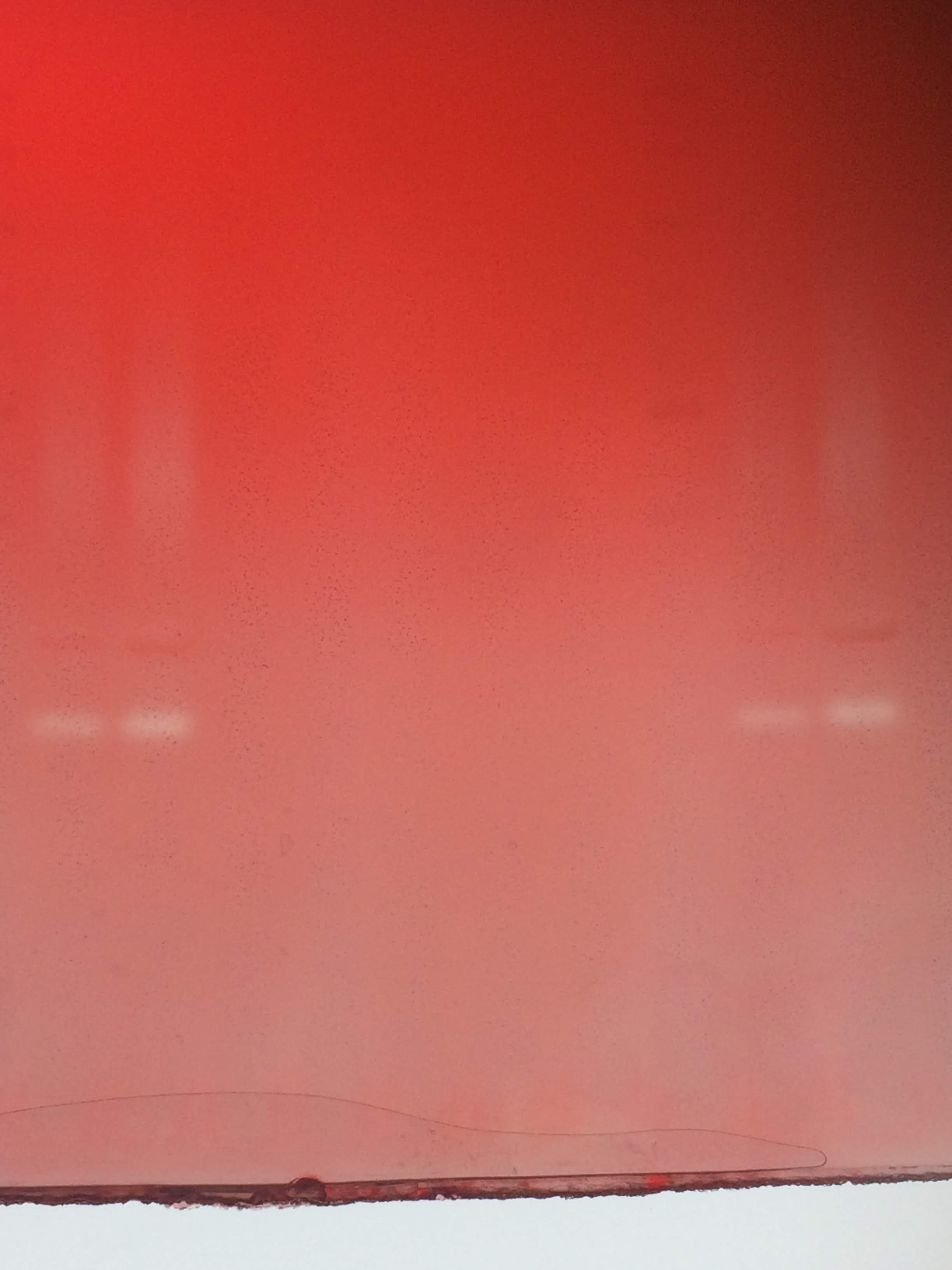


**Figure S2**

*Zymogram of the partially purified xylanase enzyme*
